# Antioxidant Capacities of Hot Water Extracts and Endopolysaccharides of Selected Chinese Medicinal Fruits

**DOI:** 10.3390/cancers8030033

**Published:** 2016-03-09

**Authors:** Sang Chul Jeong, Ratna Tulasi, Sundar Rao Koyyalamudi

**Affiliations:** 1 School of Science and Health, University of Western Sydney, Locked Bag 1797, Penrith South DC, NSW 1797, Australia; j1685@nnibr.re.kr; 2 Freshwater Bioresources Utilization Division, Nakdonggang National Institute of Biological Resources, Sangju-si 37242, Korea; 3 Department of Nuclear Medicine, Prince of Wales Hospital, Randwick, Sydney, NSW 2031, Australia; ratna.tulasi@yahoo.com; 4 Institute of Endocrinology and Diabetes, The Children’s Hospital at Westmead, Sydney, NSW 2145, Australia; 5 Discipline of Paediatrics and Child Health, The Children’s Hospital at Westmead, The University of Sydney, Sydney, NSW 2145, Australia

**Keywords:** fruits, phenols, flavonoids, polysaccharides, anti-oxidant activity

## Abstract

Fruits are a rich source of antioxidants and traditional Chinese fruits have been studied for their chemopreventive and chemotherapeutic properties against cancers and other diseases. The total phenol and flavonoid contents of eleven Chinese fruits extracts were determined. Total phenolic and flavonoid contents were estimated by both the Folin-Ciocalteau and aluminium chloride methods. The antioxidant activities were evaluated by four assays: a biological assay using *Saccharomyces cerevisiae*, DPPH radical scavenging activity, chelating ability for ferrous ions and ferric reducing antioxidant power (FRAP). The phenols and flavonoids contents of the hot water extracts were in the range of 17.7 to 94.7 mg/g and 12.3 to 295.4 mg/g, whereas the endopolysaccharides lie in the range of 4.5 to 77.4 mg/g and 22.7 to 230.0 mg/g. Significant amounts of phenols and flavonoids were present in the majority of the fruit extracts and showed strong antioxidant activities. The antioxidant properties of the fruit extracts of *Crataegus pinnatifida*, *Illicium verum*, *Ligustrum lucidum*, *Momordica grosvenori* and *Psoralea corylifolia* as determined by the DPPH and FRAP methods, were significant compared to other fruit extracts. In the present study, we found that significant amounts of phenolic and flavonoid compounds were present in these fruit extracts and may contribute to *in vitro* antioxidant activities.

## 1. Introduction

Fruits are a rich source of antioxidants (polyphenols, vitamin C, carotenoids *etc.*) that can prevent the formation of free radicals. Free radicals and non-free radical compounds are constantly produced in the human body during cell metabolism [1] and these molecules contain oxygen. These reactive molecules are called reactive oxygen species (ROS). Free radicals play an important role in cell metabolism [2] and excess production of these free radicals is toxic and may cause oxidative damage to biological molecules in the human body [3,4]. There is an evidence to suggest that reactive oxygen species are involved in the pathogenesis of many diseases including age-related disorders, cancer, atherosclerosis, neurodegenerative diseases and inflammation [5,6,7,8,9]. The antioxidant compounds [9,10] can inhibit the production of ROS in the human body as well as prevent the damage of the cells caused by oxidative stress [11,12]. There is strong evidence from studies in epidemiology, animal tumor models and *in vitro* studies demonstrating that a significant number of natural compounds found in fruit and vegetables could lower the incidence of cancer [13,14,15]. Several papers have reported that phenolics and flavonoids of plants and fruits [4,9,16,17,18] show different antioxidant activities depending on the availability of their phenol and flavonoid contents. The phenolics and flavonoids have also been shown to inhibit the inflammation process and are expected to have anti-inflammatory properties. [19]. The main objectives of the present study was to (i) determine the phenolic and flavonoid contents, (ii) systematically evaluate the antioxidant activities by using *Saccharomyces cerevisiae,* DPPH radical scavenging activity, chelating ability for ferrous ions and ferric reducing antioxidant power (FRAP) and (iii) correlate their relationship with total phenolic and flavonoid content of 11 Chinese medicinal fruits. The reported medicinal uses of these fruits are given in Table 1.

## 2. Results and Discussion

### 2.1. The Yields, Total Phenolic and Flavonoid Content of HWEs and ENPs

**Figure 1:**
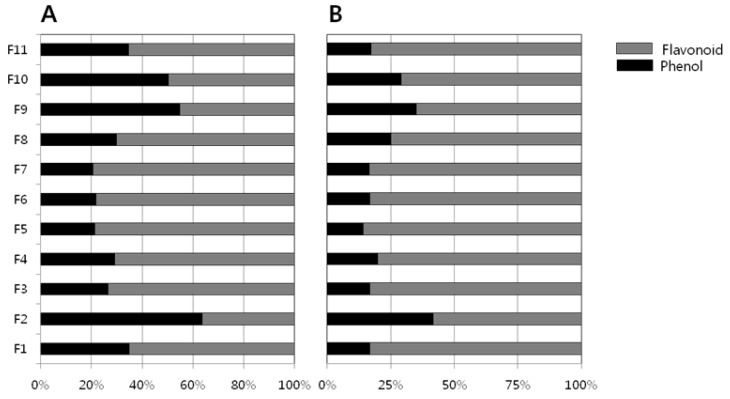
Proportional relation (%) of flavonoids content to phenolic content in the fruit extracts. (**A**) Hot water extracts; (**B**) Endopolysaccharides.

Table 2 shows the yield of hot water extracts (HWEs) and endopolysaccharides (ENPs). The yields of the HWEs are in the range 478.8 to 4580.6 mg/10 g dry fruit, whereas the ENPs lie in the range of 116.3 to 761.5 mg/10 g. The total phenolic and flavonoid content of the HWEs and ENPs were measured using the F-C reagent and aluminium chloride methods respectively. The results obtained for the HWEs and ENPs are presented in Table 3. Among the eleven Chinese medicinal fruits studied, *L. lucidum* HWE has the largest phenolic content (94 mg/g) followed by *A. tsao-ko* (76 mg/g). The ENP of *L. lucidum* contained significant amount of phenolic compounds (77 mg/g) followed by *M. grosvenori* (52 mg/g) and *P. corylifolia* (53 mg/g) ENPs. Other fruits ENPs contained appreciable amounts of phenolic compounds in the range of 21–37 mg except *A. tsao-ko* (4.5 mg/g). The amounts of flavonoid contents in majority of the fruit extracts (HWEs and ENPs) have the largest values compare to phenolic contents (Table 3). The proportional relation (%) of flavonoid contents to phenolic contents in the present study fruit extract were shown in Figure 1. The flavonoid contents were the chief constituents (75%) in most of the fruit except *Lycium barbarum* and *Momordica grosvenori* fruits (Figure 1) where the phenolic contents were present more than 25%.

### 2.2. Anti-Oxidant Activities

The antioxidant activities of eleven selected medicinal fruits were evaluated by four methods, namely, DPPH free radical scavenging, ferrous ion-chelating capacity, ferric-reducing antioxidant power (FRAP) and yeast based antioxidant screening assay. The results are presented in Table 3. The majority of the hot water extracts (HWEs) and endopolysaccharides (ENPs) showed antioxidant activity against yeast model on the basis of inhibition ability against oxidative stress induced by H_2_O_2_ except *I. verum* and *P. corylifolia* fruits. The mechanism of antioxidant activity of fruit extracts possibly a direct inactivating effect on H_2_O_2_ by scavenging hydroxyl radicals or an activation of the cellular oxidative response, which would allow increased yeast growth [40].

Most of the fruits showed significant scavenging capacity and it was moderately varied between HWEs to ENPs. The DPPH radical absorbs at 515 nm and this absorption is inhibited in the presence of antioxidants such as phenolics and flavonoids. The percentage of DPPH inhibition in HWEs ranged from 26.37% to 77.40% while in ENPs it ranged from 4.45% to 76.37% (Table 3). The highest DPPH inhibition was shown by *L. lucidum* and the lowest by *S. chinensis*. The other fruits showed significant scavenging activity against DPPH in both HWEs and ENPs. The results in this study revealed that the extracts obtained from these fruits was free radical scavengers which reacted with DPPH radical by their electron donating ability [39] and their antioxidant activities due to a combination of their total phenolic and flavonoid content as reported in other studies [41,42].

The ability of the extracts to bind with Fe^2+^ in the presence of ferrozine is compared with that of EDTA, which is a strong chelating agent that binds to metals via four carboxylate and two amine groups [43]. The ferrous ion-chelating ability results are summarised in Table 3. Among the eleven fruits studied, five of them (*Prunus mume*, *C. pinnatifida*, *I. verum*, *M. grosvenori* and *Schisandra chinensis*) do not show chelating capacity. The other fruit extracts showed significant capacity for Fe^2+^ binding, suggesting their antioxidant ability. The HWEs and ENPs of *Lycium barbarum*, *A. tsao-ko* and *P. corylifolia* can be interpreted as having the highest ferrous ion chelating ability among the fruits studied. Flavonoid and phenolic compounds are known to act as antioxidants, radical scavengers and metal chelators [44].

The antioxidant activity of the HWEs and ENPs measured by the FRAP assay are given in Table 3. The FRAP activity is significant in some of the analysed samples due to the presence of phenolic and flavonoid compounds and a similar trend is observed for many other plant extracts that have been studied [45]. The reducing power of a compound is dependents on its electron transfer ability [46]. The HWEs of *I. verum*, *A. tsao-ko* and *L. lucidum* exhibited high DPPH and FRAP values which can be interpreted as the highest antioxidant activities of the fruits studied. Also these three fruit extracts contained high content of phenolics and flavonoids. The ENPs of *L. lucidum* and *C. pinnatifida* showed significant DPPH and FRAP activities compare to other fruit ENPs. The fruit extracts of *L. lucidum* and *C. pinnatifida* had stronger DPPH radical scavenging activity and these fruit extracts are using for the treatment of different disorders [28,32]. The fruit extracts of *L. lucidum* showed protective effect against BHT-induced oxidative stress in rats [47]. The fruit extract of *C. pinnatifida* showed good antioxidant activity and neuroprotective effects [48].

### 2.3. Correlation between Antioxidant Capacities and Total Phenolic and Flavonoid Content

**Figure 2:**
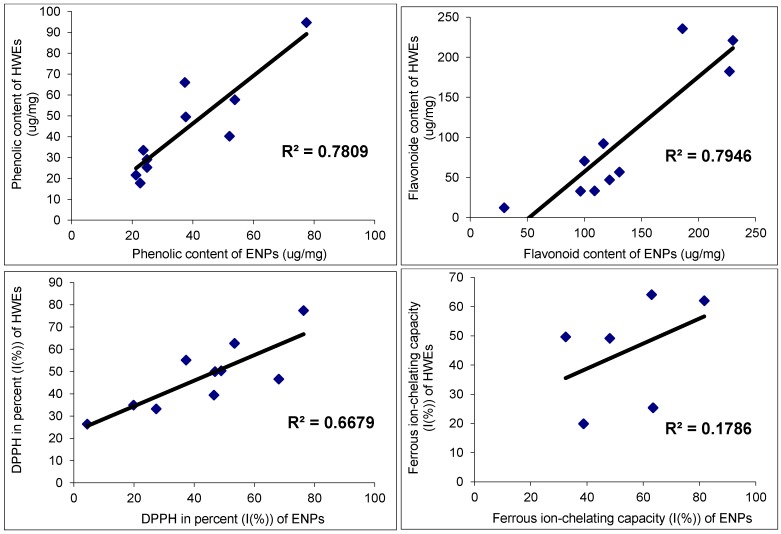
Correlation R^2^ values of phenolic and flavonoids contents, and DPPH and ferrous ion-chealting activities between the endopolysaccharides and hot water extracts.

**Figure 3:**
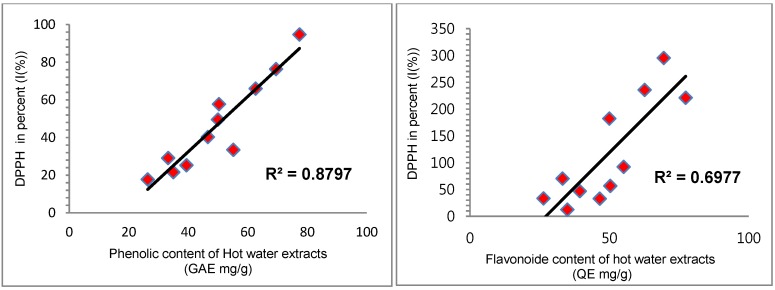

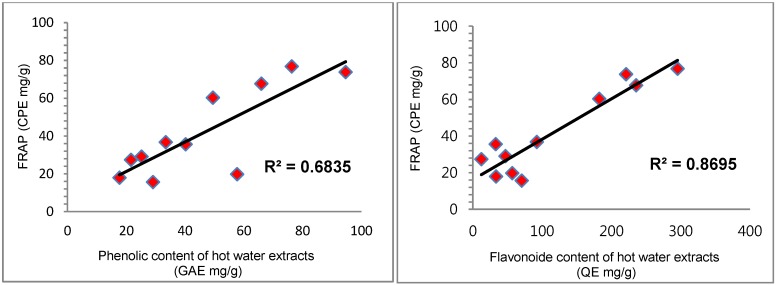
Correlation R^2^ values of antioxidant activities between phenolic and flavonoid contents (hot water extracts).

**Figure 4:**
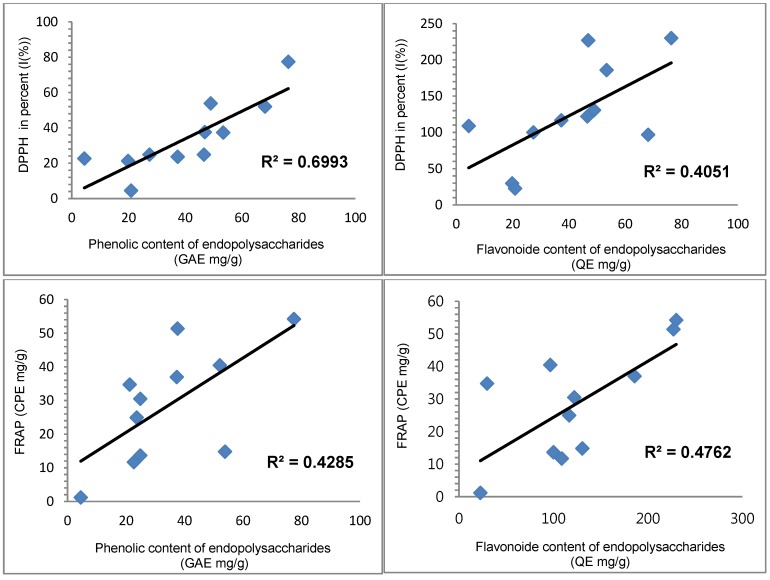
Correlation R^2^ values of antioxidant activities between phenolic and flavonoid contents (endopolysaccharides).

The correlation between the phenolic and flavonoid content, and their antioxidant activities were studied. The correlation results are presented in Table 4 and Figure 2, Figure 3 and Figure 4. The antioxidant activities of the present study showed a significant correlation with phenolics and flavonoids except ferrous-chelating capacity. The correlation with hot water extracts were: R^2^ = 0.8797 between DPPH and total phenolics, R^2^ = 0.6977 between DPPH and flavonoids, R^2^ = 0.6835 between FRAP and total phenolics and R^2^ = 0.8695 between FRAP and flavonoids. The correlations with endopolysaccharides (ENPs) were: R^2^ = 0.6993 between DPPH and total phenolics, R^2^ = 0.4051 between DPPH and flavonoids, R^2^ = 0.4285 between FRAP and phenolics and R^2^ = 0.4762 between FRAP and flavonoids, respectively. The significant correlations between the antioxidant activities and phenolics & flavonoids showed that the phenolics and flavonoids largely contribute to the antioxidant activities of these fruit extracts, and could play an important role in biological activities. The antioxidant activities were significant in hot water extracts compare to endopolysaccharides. In addition, a good linear relationship was noticed in hot water extracts among antioxidant activities of DPPH and FRAP.

## 3. Experimental Section

### 3.1. Fruits

The dried fruits were obtained from Beijing Tong Ren Tang Chinese Herbal Medicine shop, Sydney, Australia. A voucher specimen of each sample has been deposited in the University laboratory. The scientific names and family names are given in Table 1. The samples were ground to a fine powder in a grinder before extraction.

### 3.2. Chemicals and Reagents 

Gallic acid, quercetin, DPPH, DMSO, sodium carbonate, aluminium chloride, sodium nitrate, sodium hydroxide, hydrogen peroxide, Folin-Ciocalteu (F-C) reagent, ascorbic acid, 95% ethanol, bovine serum albumin (BSA), lipopolysaccharide (LPS: *E. coli* serotype 0127:B8), EDTA, N-(1-1-napthyl) ethylenediamine dihydrochloride, were purchased from Sigma (Sydney, Australia) and Lomb Scientific Pty Ltd (Sydney, Australia).

### 3.3. Preparation of Hot Water Extracts and Endopolysaccharides

**Figure 5:**
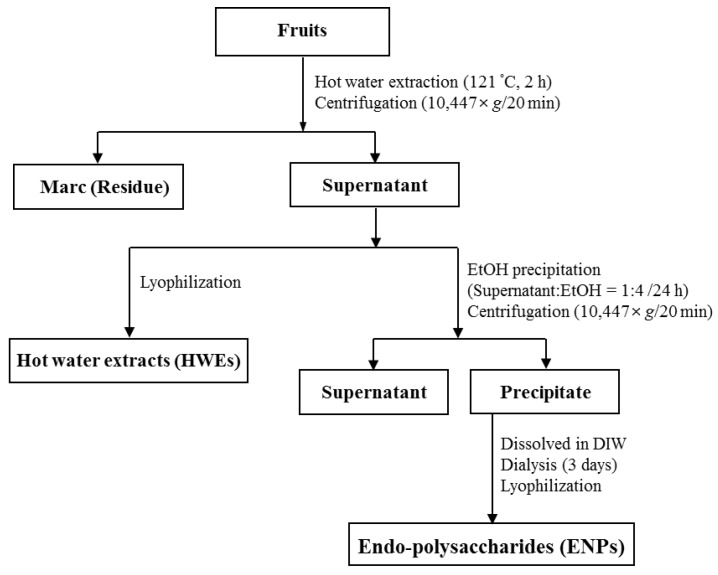
Schematic diagram for the processing of hot water extracts and endopolysaccharides from medicinal fruits.

Approximately 30 g of each ground fruit was autoclaved with 50 mL of deionised water at 121 °C for 2 h. The extracted samples were centrifuged at 10,447 *g* for 20 min and the supernatant transferred to a 50 mL volumetric flask. The residue was further rinsed two more times, and the pooled the extract adjusted to 100 mL. The preparation and isolation of hot water extracts and endopolysaccharides from the plants was as described previously [49,50,51] and is summarised in Figure 5. All hot water extracts and endopolysaccharides were filtered through a 0.45 µm nylon filter before analysis.

### 3.4. Determination of Total Phenolic Content

The total phenolic content was determined using the Folin-Ciocalteu (F-C) colorimetric method [52]. Briefly, 50 µL of sample and 50 µL of F-C reagent were pipetted into an Eppendorf tube. The contents were vortexed for 10 sec and then left to stand at room temperature for 2 min before the reaction was stopped by adding 500 µL 5% (w/v) sodium carbonate solution and 400 µL distilled water and the volume adjusted to 1 mL. The mixture was then vortexed and incubated at 45 °C for 30 min before cooling rapidly with ice. The solution absorbance was measured at 760 nm. Gallic acid concentrations ranging from 25–300 µg/mL were prepared and the calibration curve was obtained using a linear fit (R^2^ = 0.9961) [42]. The samples were analysed in duplicate.

### 3.5. Determination of Total Flavonoid Content

The total flavonoids were estimated by the aluminium chloride method [53]. Briefly, 0.5 mL of the sample and 300 µL of NaNO_2_ (1:20 w/v) were pipetted into a test tube and the contents were vortexed for 10 s and left to stand at room temperature for 5 min. After standing, 300 µL of AlCl_3_ (1:10 w/v), 2 mL of 1M NaOH and 1.9 mL of distilled water was added to the reaction mixture which was then vortexes for 10 sec and the absorbance measured at 510 nm. Quercetin concentrations ranging from 0 to 1200 µg/mL were prepared and the standard calibration curve obtained using a linear fit (R^2^ = 0.9980). The samples were analysed in duplicate.

### 3.6. Free Radical DPPH Scavenging Assay

The DPPH assay was carried out according to the procedure of Brand-William *et al*. [54], with minor modifications. In this study, different volumes (10, 20, 30, 40, 50, 60, 70, 80, 90, and 100 μL) of endopolysaccharides and hot water extracts were mixed with a methanolic solution of DPPH radical (2.2 mg/L, 200 μL) in a 96 well microplate. The final volume of each well was made up to 300 μL by adding an appropriate amount of methanol. The mixture was shaken gently on a microplate reader, and the absorbance at 515 nm taken every 2 min for 30 min or until the absorbance reached its maximum value. The DPPH concentration in the reaction medium was calculated from a calibration curve derived from a serial dilution of the DPPH standard. The control (containing all reagents except the test compound) and standards were subject to the same procedure. The free radical scavenging activity is expressed as the inhibition percentage of free radicals by the sample and calculated using following the formula:

% of DPPH radical scavenging effect = [(A_control_ – A_sample_)/A_control_] × 100

where A_control_ is the absorbance of control and A_sample_ is the absorbance of sample at 515 nm. The samples were analysed in triplicate.

### 3.7. Assay for Screening of Scavenging Activity in a 96-well Microplate Using Saccharomyces Cerevisiae

The antioxidant capacity of the fruit extracts was also measured using a *Saccharomyces cerevisiae*-based high throughput assay [4,55]. Yeast is the most characterized eukaryote at the molecular level; it serves as a paradigm for higher eukaryotes like plants and animals in fundamental cellular studies. The yeast based assay utilises the important physiological phenomenon of oxidative stress-mediated cell cycle arrest to screen the oxidant scavenging and intracellular antioxidant activity for a chemically diverse range of compounds. *Saccharomyces cerevisiae* BY4743 was cultured overnight in a 50 mL volume by inoculation of a single colony. The culture was then diluted to an optical density at 600 nm (OD_600_) of 0.2 in media, and 180 µL of the strain was added to each well in a 96-well microplate where 10 µL per well of each herbal extract had been added in duplicate. 10 µL of 32% v/v hydrogen peroxide of (H_2_O_2_) was added to give a final concentration of 4 mM. The initial OD_600_ reading was taken using a microplate reader (Multiskan EX, Thermo Electron, Waltham, MA, USA), and the plates were then incubated in a 30 °C incubator with shaking at 750 rpm. Yeast growth was monitored by reading the OD_600_ until 20 h. Ascorbate was used as the positive control and samples were analysed in duplicate.

### 3.8. Ferrous Ion-Chelating Effect

The ferrous ion-chelating effect of the sample was estimated by the method of Chua *et al.* [56]. Briefly, 740 μL of methanol and 200 μl of sample were incubated with 20 μL of 2 mM FeCl_2_ solution. The reaction was initiated by adding 40 μL of 5 mM ferrozine into the mixture which was then allowed to stand at ambient temperature for 10 min. The absorbance of the reaction mixture was measured at 562 nm. Distilled water instead of ferrozine solution was used as a blank, to correct for any unequal colour of the sample solution. EDTA-Na_2_ was used as a reference standard. The ferrous ion-chelating ability was calculated as follows:

Ferrous ion-chelating ability (%) = [A_control_ − (A_sample_ − A_blank_)]/A_control_ × 100

where A_control_ is the absorbance of the control, A_sample_ the absorbance of sample or standard and A_blank_ the absorbance of the blank.

### 3.9. Ferric-Reducing Antioxidant Power Assay (FRAP)

The ferric-reducing antioxidant power (FRAP) of the extracts was tested using the assay of Oyaizu [57]. One mL of different concentrations of the samples as well as the reference chlorogenic acid was added to 2.5 mL phosphate buffer (0.1 M, pH 6.6) and 2.5 mL potassium ferricyanide (1.0%, w/v). Later, each mixture was incubated at 50 °C for 20 min and then 2.5 mL 10% trichloroacetic acid added. After the mixture was shaken vigorously, 2.5 mL of the solution was mixed with 2.5 mL distilled water and 0.5 mL of FeCl_3_ (0.1%, w/v). After 30 min incubation, absorbances were read at 700 nm. Each analysis was carried out in duplicate. Increased absorbance of the reaction mixture indicates increased reducing power.

### 3.10. Data Presentation and Analysis

The results are expressed as mean ± standard deviation, calculated from the duplicate determinations. The linear relationships and significance tests of these data sets were also calculated. GraphPad Prism 5.01 (GraphPad Software, Inc., La Jolla, CA, USA) was used for the growth curve analysis of the dose-dependent experiments.

## 4. Conclusions

In general, fruits contain several nutrients such as fibres, micronutrients and vitamins. Moreover, secondary metabolites were identified in fruits, especially phenolic compounds. The phenolic compounds in fruits are the chief bioactive compounds and known for several health benefits. The antioxidant activities and antioxidant contents (phenolics and flavonoids) of 11 selected medicinal fruits were evaluated. The hot water extracts and endopolysaccharides obtained from the fruits in present study showed significant antioxidant properties. Some of the fruit extracts showed a strong correlation between DPPH and FRAP value which suggests that antioxidants in these HWEs and ENPs possess free radical scavenging activity and oxidant reducing power. The present study results show further evidence about the importance of phenolic compounds and their antioxidant activities. The fruit extracts in the present study showed moderate antioxidant activities and could be used in herbal formulations for the prevention of diseases caused by free radicals in biological systems like cancer. These fruits could be considered as antioxidant and cancer therapeutic agents. Further research is however needed to establish health benefits of these fruit extracts.

## Figures and Tables

**Table 1:** List of medicinal fruits used in this study.

Chinese Fruits	Family Name	Medicinal Uses
1. *Prunus mume* Siebold & Zucc.	Rosaceae	Fever, cough and intestinal disorders [20]
2. *Lycium barbarum* L.	Solanaceae	Cardiovascular and inflammatory diseases [21], vision-related diseases [22], such as age-related macular degeneration and glaucoma [23] or from neuroprotective [24], anticancer [25] or immunomodulatory activity.
3. *Amomum villosum* Lour.	Zingiberaceae	Digestion disorder [26]
4. *Amomum kravanh* Pirre ex Gagnep.	Zingiberaceae	Anti-asthmatic [27]
5. *Crataegus pinnatifida* Bunge	Rosaceae	Anticancer [28]
6. *Illicium verum* Hook. f.	lliciaceae	Antibacterial [29], neurotropic, hypothermic and analgesic [30]
7. *Amomun tsao-ko* Crevost & Lemarié	Zingiberaceae	Malaria, throat infections, abdominal pain, stomach disorders, dyspepsia, nausea, vomiting and diarrhea [31]
8. *Ligustrum lucidum* W.T.Aiton	Oleaceae	Immunomodulatory, anti-inflammatory, hepatoprotective, anti-tumor and anti-aging act [32]
9. *Momordica grosvenori* Swingle	Curcubitaceae	Anti-diabetic [33], anticancer [34], anti-inflammatory, antioxidant, anti-diabetic, and nephroprotective properties [35]
10. *Psoralea corylifolia* L.	Fabaceae	Anticancer [36,37]
11. *Schisandra chinensis* Turcz. Baill.	Schisandraceae	Anti-inflammatory [38], antiviral, anticancer and neuroprotective effects [39]

**Table 2:** Yields of hot water extracts and endopolysaccharides of fruits studied.

Chinese Fruits	Hot Water Extracts (mg/10 g Dry Fruit)	Endopolysaccharides (mg/10 g Dry Fruit)
1. *Prunus mume*	2864.40	500.18
2. *Lycium barbarum*	4580.86	200.50
3. *Amomum villosum*	887.07	446.86
4. *Amomum kravanh*	478.81	240.99
5. *Crataegus pinnatifida*	3239.41	761.56
6. *Illicium verum*	1440.41	387.42
7. *Amomun tsao-ko*	514.61	116.34
8. *Ligustrum lucidum*	1601.63	397.34
9. *Momordica grosvenori*	2737.64	365.29
10. *Psoralea corylifolia*	1404.95	263.80
11. *Schisandra chinensis*	3250.40	474.40

**Table 3:** Antioxidant activities of endopolysaccharides (ENPs) and hot water extracts (HWEs) obtained for Chinese fruits studied.

Chinese Fruits	Scavenging Activity on Yeast	Total Phenol Content (GAE mg/g ± SD)	Total Flavonoid Content (QE mg/g ± SD)	DPPH in Percent (*I*(%)) ^a^	Ferrous Ion-Chelating Ability		Ferric-Reducing Antioxidant Power (CPE ^c^ mg/g ± SD)
					% ^b^ ± SD	EDTA Equivalent (ug/mL ± SD)	
1-ENP ^d^	+ ^f^	24.85 ± 0.52	122.06 ± 3.39	46.58 ± 0.00	-	-	30.48 ± 0.11
2-ENP	+	21.24 ± 0.39	29.76 ± 0.99	19.86 ± 0.97	62.98 ± 0.37	226.91 ± 1.93	34.73 ± 0.25
3-ENP	+	23.65 ± 0.39	116.66 ± 1.41	37.33 ± 0.48	63.50 ± 2.34	229.64 ± 12.21	24.91 ± 0.42
4-ENP	+	24.85 ± 0.52	100.06 ± 2.55	27.40 ± 0.97	32.40 ± 5.42	67.36 ± 28.28	13.63 ± 0.11
5-ENP	+	37.63 ± 0.26	227.06 ± 2.83	46.92 ± 0.48	-	-	51.36 ± 0.21
6-ENP	−	37.35 ± 0.13	185.96 ± 0.42	53.42 ± 0.97	-	-	36.98 ± 0.39
7-ENP	− ^g^	4.51 ± 0.09	22.66 ± 0.57	20.89 ± 0.48	81.71 ± 1.72	324.64 ± 9.00	1.16 ± 0.07
8-ENP	+	77.44 ± 0.26	230.06 ± 0.57	76.37 ± 1.45	38.76 ± 2.09	100.55 ± 10.93	54.21 ± 0.14
9-ENP	+	52.07 ± 0.26	96.66 ± 0.85	68.15 ± 1.45	-	-	40.46 ± 0.35
10-ENP	−	53.83 ± 0.39	130.66 ± 2.55	48.97 ± 1.45	48.08 ± 0.74	149.18 ± 3.86	14.78 ± 0.32
11-ENP	+	22.63 ± 0.26	108.86 ± 0.57	4.45 ± 27.61	-	-	11.68 ± 0.11
1-HWE ^e^	+	25.22 ± 0.26	47.10 ± 0.94	39.38 ± 0.48	-	-	29.03 ± 0.25
2-HWE	+	21.61 ± 0.13	12.35 ± 0.82	34.93 ± 0.97	64.11 ± 1.48	232.82 ± 7.71	27.33 ± 0.53
3-HWE	+	33.46 ± 0.39	92.27 ± 1.18	55.14 ± 1.45	25.35 ± 0.37	30.55 ± 1.93	36.71 ± 0.71
4-HWE	+	29.11 ± 0.26	70.52 ± 4.83	33.22 ± 1.45	49.65 ± 1.23	157.36 ± 6.43	15.61 ± 0.21
5-HWE	+	49.48 ± 0.26	182.40 ± 5.66	50.00 ± 0.00	-	-	60.28 ± 1.31
6-HWE	−	65.96 ± 1.31	235.73 ± 0.94	62.67 ± 0.48	-	-	67.71 ± 0.71
7-HWE	+	76.33 ± 2.62	295.40 ± 0.47	69.52 ± 0.48	62.02 ± 5.17	221.91 ± 27.00	76.78 ± 0.60
8-HWE	+	94.67 ± 1.31	221.07 ± 3.77	77.40 ± 0.97	19.86 ± 9.12	1.91 ± 47.57	73.78 ± 0.18
9-HWE	+	40.22 ± 1.31	33.02 ± 0.12	46.58 ± 0.00	-	-	35.56 ± 0.28
10-HWE	−	57.72 ± 0.65	56.85 ± 3.89	50.34 ± 0.48	49.13 ± 2.96	154.64 ± 15.43	19.76 ± 0.21
11-HWE	+	17.72 ± 0.39	33.43 ± 0.24	26.37 ± 2.42	-	-	17.93 ± 0.39

^a^ The ratio of inhibition of free radical by DPPH in percent (*I*(%)) as follows; *I* (%) = [(A_blank_ − A_sample_)/A_blank_] × 100; ^b^ Ferrous ion-chelating ability as follows; % = [A_control_ − (A_sample_ − A_blank_)]/A_control_ × 100; ^c^ Ferric reducing antioxidant power was expressed in g of chlorogenic acid power (CPE) per mg of dry weight; ^d^ ENP: endopolysaccharide; ^e^ HWE: hot water extract; ^f^ positive activity; ^g^ no activity; Yeast oxidative stress was measure on the basis of survival of yeast cells (yeast growth) after treatment of H_2_O_2_.

**Table 4:** Correlation R^2^ values between the chemical compound and antioxidant activities of Chinese fruits.

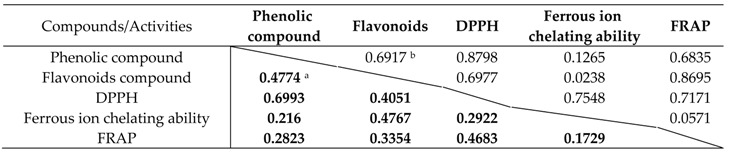

^a^ Bold font values are correlation R2 values of endopolysaccharides; ^b^ Normal font value are correlation R2 values of hot water extracts.

## References

[1] Bloknina O., Virolainen E., Fagerstedt K.V. (2003). Antioxidant, oxidative damage and oxygen deprivation studies: A review. Ann. Bot..

[2] Ajith T.A., Janardhanan K.K. (2007). Indian medicinal mushrooms as a source of antioxidant and antitumor agents. J. Clin. Biochem. Nutr..

[3] Liu H., Visner G.A., Dani S.Z., Helmut H., Jaishree J., Abida K.H., Philip T.C., Roberto B. (2008). Oxidants and antioxidants. Molecular Pathology of Lung Diseases.

[4] Zhang L., Ravipati A.S., Koyyalamudi S.R., Jeong S.C., Reddy N., Smith P.T., Bartlett J., Shanmugam K., Münch D.G., Wu M.J. (2011). Antioxidant and anti-inflammatory activities of selected medicinal plants containing phenolic and flavonoid compounds. J. Agric. Food Chem..

[5] Diaz M.N., Frei B., Vita J.E., Keaney J.F. (1997). Antioxidants and atherosclerotic heart disease. N. Engl. J. Med..

[6] Aruoma O.I. (1998). Free radicals, oxidative stress and antioxidants in human health and disease. J. Am. Oil Chem. Soc..

[7] Burns J., Gardner P.T., Matthew D., Duthie C.G., Lean M.E., Crozier A. (2001). Extraction of phenolics and changes in antioxidant activity of red wines during vinification. J. Agric. Food Chem..

[8] Tang S.Y., Whiteman M., Peng Z.F., Jenner A., Yong E.L., Halliwell B. (2004). Characterization of antioxidant and antiglycation properties and isolation of active ingredients from traditional Chinese medicines. Free Radic. Biol. Med..

[9] Cai Y., Luo Q., Sun M., Corke H. (2004). Antioxidant activity and phenolic compounds of 112 traditional Chinese medicinal plants associated with anticancer. Life Sci..

[10] Dragland S., Senoo H., Wake K., Holte K., Blomhoff R. (2003). Several culinary and medicinal herbs are important sources of dietary antioxidants. J. Nutr..

[11] Akinmoladun A.C., Obuotor E.M., Farombi E.O. (2010). Evaluation of antioxidant and free radical scavenging capacities of some Nigerian indigenous medicinal plants. J. Med. Food.

[12] Özen T., Çöllü Z., Korkmaz H. (2010). Antioxidant Properties of Urtica pilulifera Root, Seed, Flower, and Leaf Extract. J. Med. Food.

[13] Riboli E., Norat T. (2003). Epidemiologic Evidence of the protective effect of fruit and vegetables on cancer risk. Am. J. Clin. Nutr..

[14] Tadjalli-Mehr K., Becker N., Rahu M., Stengrevics A., Kurtinaitis J., Hakama M. (2003). Randomized trial with fruits and vegetables in prevention of cancer. Acta Oncol..

[15] Temple N.J., Gladwin K.K. (2003). Fruit, vegetables, and the prevention of cancer: Research challenges. Nutrition.

[16] Hendra R., Ahmad S., Oskoueian E., Sukari A., Shukor M.Y. (2011). Antioxidant, Anti-inflammatory and Cytotoxicity of *Phaleria macrocarpa* (Boerl.) Scheff Fruit. BMC Complement. BMC Complement. Altern. Med..

[17] Diaz P., Jeong S.C., Lee S., Khoo C., Koyyalamudi S. (2012). Antioxidant and anti-inflammatory activities of selected medicinal plants and fungi containing phenolics and flavonoid compounds. Chin. Med. J..

[18] Ravipati A.S., Zang L., Koyyalamudi S., Reddy N., Bartlett J., Smith P., Shanmugam K., Munch G., Wu M.J. (2012). Antioxidant and anti-inflammatory activities of selected Chinese medicinal plants and their relation with antioxidant content. BMC Complement. Altern. Med..

[19] Talhouk R., Karam C., Fostok S., El-Jouni W., Barbour E. (2007). Anti-inflammatory bioactivities in plant extracts. J. Med. Food.

[20] Jo S.C., Nam K.C., Min B.R., Ahn D.U., Cho S.H., Park W.P., Lee S.C. (2006). Antioxidant activity of *Prunus mume* extract in cooked chicken breast meat. Int. J. Food Sci. Tech..

[21] Luo Q., Li Z., Huang X., Yan J., Zhang S., Cai Y.Z. (2006). Lycium barbarum polysaccharides: Protective effects against heat-induced damage of rat testes and H2O2-induced DNA damage in mouse testicular cells and beneficial effect on sexual behavior and reproductive function of hemicastrated rats. Life Sci..

[22] Cheng C.Y., Chung W.Y., Szeto Y.T., Benzie I.F. (2005). Fasting plasma zeaxanthin response to *Fructus barbarum* L. (wolfberry; Kei Tze) in a food-based human supplementation trial. Br. J. Nutr..

[23] Chan H.C., Chang R.C., Koon-Ching Ip.A., Chiu K., Yuen W.H., Zee S.Y., So K.F. (2007). Neuroprotective effects of *Lycium barbarum* Lynn on protecting retinal ganglion cells in an ocular-hypertension model of glaucoma. Exp. Neurol..

[24] Yu M.S., Leung S.K., Lai S.W., Che C.M., Zee S.Y., So K.F., Yuen W.H., Chang R.C. (2005). Neuroprotective effects of anti-aging oriental medicine *Lycium barbarum* against beta-amyloid peptide neurotoxicity. Exp. Gerontol..

[25] Gan L., Hua Z.S., Liang Y.X., Bi X.H. (2004). Immunomodulation and antitumor activity by a polysaccharide-protein complex from *Lycium barbarum*. Int. Immunopharmacol..

[26] Zhu J.Z., Zhang J., Zhang Z.J., Wang W. (2006). Effects of *Amomum villosum* on functional digestion disorder in rats. West Chin. J. Pharm. Sci..

[27] Lee M., Lee N., Lee J., Jung D., Lee H., Seo C., Kim J., Kim J., Ha H., Shin H. (2010). Anti-asthmatic Effects of *Amomum compactum*. J. Allergy Clin. Immunol..

[28] Kao E.S., Wang C.J., Lin W.L., Chu C.Y., Tseng T.H. (2007). Effects of polyphenols derived from fruit of *Crataegus pinnatifida* on cell transformation, dermal edema and skin tumor formation by phorbol ester application. Food Chem. Toxicol..

[29] Yang J.F., Yang C.H., Chang H.W., Yang C.S., Wang S.M., Hsieh M.C., Chuang L.Y. (2010). Chemical composition and antibacterial activities of *Illicium verum* against antibiotic-resistant pathogens. J. Med. Food.

[30] Nakamura T., Okuyama E., Yamazaki M. (1996). Neurotropic components from star anise (Illicium verum Hook. fil.). Chem. Pharm. Bull..

[31] Jiangsu New Medical College (1977). Dictionary of Chinese Materia Medical.

[32] He Z.D., Dong H., Xu H.X., Ye W.C., Sun H.D., But P.P.H. (2001). Secoiridoid constituents from the fruits of *Ligustrum lucidum*. Phytochemistry.

[33] Song F., Qi X., Chen W., Jia W., Yao P., Nussler A.K., Sun X., Liu L. (2007). Effect of *Momordica grosvenori* on oxidative stress pathways in renal mitochondria of normal and alloxan-induced diabetic mice. Involvement of heme oxygenase-1. Eur. J. Nutr..

[34] Takasaki M., Konoshima T., Murata Y., Sugiura M., Nishino H., Tokuda H., Matsumoto K., Kasai R., Yamasaki K. (2003). Anticarcinogenic activity of natural sweeteners, cucurbitane glycosides, from *Momordica grosvenori*. Cancer Lett..

[35] Song F., Chen W., Jia W., Yao P., Nussler A.K., Sun X., Liu L. (2006). A natural sweetener, Momordica grosvenori, attenuates the imbalance of cellular immune functions in alloxaninduced diabetic mice. Phytother. Res..

[36] Szliszka E., Czuba Z.P., Sędek L., Paradysz A., Król W. (2011). Enhanced TRAIL-mediated apoptosis in prostate cancer cells by the bioactive compounds neobavaisoflavone and psoralidin isolated from *Psoralea corylifolia*. Pharmacol. Rep..

[37] Tang S.Y., Gruber J., Wong K.P., Halliwell B. (2007). *Psoralea corylifolia* L. inhibits mitochondrial complex I and proteasome activities in SH-SY5Y cells. Ann. NY Acad. Sci..

[38] Guo L.Y., Hung T.M., Bae K.H., Shin E.M., Zhou H.Y., Hong Y.N., Kang S.S., Kim H.P., Kim Y.S. (2008). Anti-inflammatory effects of schisandrin isolated from the fruit of *Schisandra chinensis* Baill. Eur. J. Pharm..

[39] Kim S.J., Min H.Y., Lee E.J., Kim Y.S., Bae K., Kang S.S., Lee S.K. (2010). Growth inhibition and cell cycle arrest in the G0/G1 by schizandrin, a dibenzocyclooctadiene lignan isolated from *Schisandra chinensis*, on T47D human breast cancer cells. Phytothe. Res..

[40] Azevedo F., Marques F., Fokt H., Oliveira R., Johansson B. (2011). Measuring oxidative DNA damage and DNA repair using the yeast comet assay. Yeast.

[41] Baumann J., Wurn G., Bruchlausen V. (1979). Prostaglandin synthetase inhibiting O2-Radical scavenging properties of some flavonoids and related phenolic compounds. Naunyyn-Schmiedebergs. Arch. Pharmacol..

[42] Cai Y., Sun M., Xing J., Corke H. (2004). Antioxidant phenolic constituents in roots of *Rheum officinale* and *Rubia cordifolia*: Structure-radical scavenging activity relationships. J. Agri. Food Chem..

[43] Chang H.F., Yang L.L. (2012). Radical-scavenging and rat liver mitochondria lipid peroxidative inhibitory effects of natural flavonoids from traditional medicinal herbs. J. Med. Plants Res..

[44] Mira L., Fernandez M.T., Santos M., Rocha R., Florencio M.H., Jennings K.R. (2002). Interactions of flavonoids with iron and copper ions: A mechanism for their antioxidant activity. Free Radic. Res..

[45] Katalinic V., Milos M., Kulisic T., Jukic M. (2006). Screening of 70 medicinal plant extracts for antioxidant capacity and total phenols. Food Chem..

[46] Gordon M.H., Hudson B.J.F. (1990). The Mechanism of Antioxidant Action *in vitro*. Food antioxidants.

[47] Lin H.M., Yen F.L., Ng L.T., Lin C.C. (2007). Protective effects of *Ligustrum lucidum* fruit extract on acute butylated hydroxytoluene-induced oxidative stress in rats. J. Ethanopharmacol..

[48] Chang C.L., Chen H.S., Chen Y.C., Lai G.H., Lin P.K., Wang C.M. (2013). Phytochemical composition, antioxidant activity and neuroprotective effect of *Crataegus pinnatifida* fruit. S. Afr. J. Bot..

[49] Jeong S.C., Yang B.K., Jeong Y.T., Sundar Rao K., Song C.H. (2007). Isolation and characterization of biopolymers extracted from the bark of *Acanthopanax sessiliflorus* and their anti-complement activity. J. Microbiol. Biotechnol..

[50] Zhang L., Koyyalamudi S., Jeong S.C., Reddy N., Smith P.T., Ananthan R., Longvah T. (2012). Antioxidant and immunomodulatory activities of polysaccharides from the roots of Sanguisorba officinalis. Int. J. Biol. Macromol..

[51] Jeong S.C., Koyyalamudi S., Hughes J.M., Khoo C., Bailey T., Park J.P., Song C.H. (2013). Modulation of cytokine production and complement activity bybiopolymers extracted from medicinal plants. Phytopharmacology.

[52] Cicco N., Lanorte M.T., Paraggio M., Viggiano M., Lattanzio V.A. (2009). Reproducible, rapid and inexpensive Folin-Ciocalteu micro-method in determining phenolics of plant methanol extracts. Microchem. J..

[53] Zhishen J., Mengcheng T., Jianming W. (1999). The determination of flavonoid contents in mulberry and their scavenging effects on superoxide radicals. Food Chem..

[54] Brand-Williams W., Cuveleir M.E., Berset C. (1995). Use of a free radical method to evaluate antioxidant activity. LWT-Food Sci. Technol..

[55] Wu M.J., O'Doherty P.J., Fernandez H.R., Lyons V., Rogers P.J., Dawes I.W., Higgins V.J. (2011). An antioxidant screening assay based on oxidant induced growth arrest in Saccharomyces cerevisiae. FEMS Yeast Res..

[56] Chua M.T., Tung Y.T., Chang S.T. (2008). Antioxidant activities of ethanolic extracts from twigs of *Cinnamomum osmophleum*. Bioresour. Technol..

[57] Oyaizu M. (1986). Studies on product of browning reaction prepared from glucosamine. Jpn. J. Nutr..

